# Decoding diversity in a coral reef fish species complex with restricted range using metagenomic sequencing of gut contents

**DOI:** 10.1002/ece3.6138

**Published:** 2020-03-10

**Authors:** Beverly J. French, Yan Wei Lim, Brian J. Zgliczynski, Robert A. Edwards, Forest Rohwer, Stuart A. Sandin

**Affiliations:** ^1^ Center for Marine Biodiversity and Conservation Scripps Institution of Oceanography University of California San Diego CA USA; ^2^ Department of Biology San Diego State University San Diego CA USA; ^3^ Department of Computer Science San Diego State University San Diego CA USA

**Keywords:** biodiversity, coexistence, hawkfish, metagenomics, rare species, trophic ecology

## Abstract

**Aim:**

Identification of the processes that generate and maintain species diversity within the same region can provide insight into biogeographic patterns at broader spatiotemporal scales. Hawkfishes in the genus *Paracirrhites* are a unique taxon to explore with respect to niche differentiation, exhibiting diagnostic differences in coloration, and an apparent center of distribution outside of the Indo–Malay–Philippine (IMP) biodiversity hotspot for coral reef fishes. Our aim is to use next‐generation sequencing methods to leverage samples of a taxon at their center of maximum diversity to explore phylogenetic relationships and a possible mechanism of coexistence.

**Location:**

Flint Island, Southern Line Islands, Republic of Kiribati.

**Methods:**

A comprehensive review of museum records, the primary literature, and unpublished field survey records was undertaken to determine ranges for four “arc‐eye” hawkfish species in the *Paracirrhites* species complex and a potential hybrid. Fish from four *Paracirrhites* species were collected from Flint Island in the Southern Line Islands, Republic of Kiribati. Hindgut contents were sequenced, and subsequent metagenomic analyses were used to assess the phylogenetic relatedness of the host fish, the microbiome community structure, and prey remains for each species.

**Results:**

Phylogenetic analyses conducted with recovered mitochondrial genomes revealed clustering of *P. bicolor* with *P. arcatus* and *P. xanthus* with *P. nisus*, which were unexpected on the basis of previous morphological work in this species complex. Differences in taxonomic composition of gut microbial communities and presumed prey remains indicate likely separation of foraging niches.

**Main Conclusions:**

Our findings point toward previously unidentified relationships in this cryptic species complex at its proposed center of distribution. The three species endemic to the Polynesian province (*P. nisus*, *P. xanthus*, and *P. bicolor*) cluster separately from the more broadly distributed *P. arcatus* on the basis of relative abundance of metazoan sequences in the gut (presumed prey remains)*.* Discordance between gut microbial communities and phylogeny of the host fish further reinforce the hypothesis of niche separation.

## Graphical abstract

Identification of the processes that generate and maintain species diversity within the same region can provide insight into biogeographic patterns at broader spatiotemporal scales. Here, we use next‐generation sequencing methods to leverage collections of coral reef fish species in the Paracirrhites genus to explore phylogenetic relationships and a possible mechanism of coexistence. Our findings point toward previously unidentified relationships in this cryptic species complex at its proposed center of distribution, and likely trophic partitioning on the basis of gut microbial communities and sequenced prey remains.

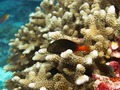

## 1 INTRODUCTION

Although multiple ecological processes for species coexistence have been proposed (Chesson, 2000), a debate remains over what processes maintain morphologically and genetically similar species in a single habitat (Siepielski & McPeek, 2010; Velland, 2010). By the principle of competitive exclusion, complete competitors should not coexist (Hardin, 1960); yet, closely related and superficially ecologically equivalent species do co‐occur in nature (Cothran, Noyes, & Relyea, 2015; Darwin, 1859; Gotelli, 1997). One mechanism contributing to initial species’ divergence and continued persistence is trophic partitioning. Trophic shifts, especially feeding niche partitioning and associated morphological changes, have frequently been invoked to explain divergence among coral reef fishes on evolutionary timescales (Bellwood, Hoey, Bellwood, & Goatley, 2014; Bellwood & Wainwright, 2006), yet current trophic designations of reef fish are coarse and do not reflect likely fine‐scale differences in diet (Sandin & Zgliczynski, 2015).

Dietary assessments are often conducted using stable isotope and gut content analysis, but these approaches have limitations. For gut content analysis, an important limitation is linked to difficulties with taxonomic identification of partially digested prey remains. Furthermore, even those identifiable remains may represent limited bouts of feeding (Baker, Buckland, & Sheaves, 2013; Dam, Peterson, & Okubo, 1991), especially for carnivorous fish species with rapid digestion processes. The accuracy of the results from stable isotopes is heavily dependent on whether sources have been accurately identified, despite advances in models incorporating uncertainty (Ward, Semmens, & Schindler, 2010).

High‐throughput sequencing techniques, including metagenomic sequencing, of coral reef fish intestines and gut homogenates provide an opportunity to close current gaps in knowledge for investigations of high‐resolution trophic partitioning among coral reef fishes. Results from metabarcoding studies have already shown promise for fishes (Casey et al., 2019; Leray, Boehm, Mills, & Meyer, 2012; Leray, Agudelo, Mills, & Meyer, 2013; Leray, Meyer, & Mills, 2015) and other species (Andriollo, Gillet, Michaux, & Ruedi, 2019; Deagle et al., 2018). However, examination of gut contents, both by traditional taxonomy and metabarcoding, provides only a snapshot of short‐term feeding behavior, making extrapolation to general ecological behaviors difficult. Metagenomic sequencing, in contrast, provides information on the microbiome in addition to possible prey sequences (Srivathsan, Sha, Vogler, & Meier, 2014). Although prey sequences suffer from the same shortcomings of both morphological gut content analysis and metabarcoding in that they are still a snapshot of last meals, and should be interpreted as such, information from the gut microbiome holds more promise. One of the most important environmental factors that influences gut microbiota is host diet (Bolnick et al., 2014; Muegge et al., 2011; Smriga, Sandin, & Azam, 2010; Sullam et al., 2012; Wong & Rawls, 2012), and in studies which have parsed the effects of diet and phylogeny, gut microbial communities cluster strongly by diet and only weakly by host species (Hale et al., 2018). In organisms including primates and fish, distinct gut microbiomes are associated with different diets (Hale et al., 2018; Uchii et al., 2006, Scott, Gratz, Sheridan, Flint, & Duncan, 2013), providing strong evidence that the gut microbiome can allow us to distinguish feeding habits within closely related species. For example, fish of the same species with different feeding habits (*Lepomis macrochirus*) have been shown to have distinct intestinal microbiota associated with different feeding modes, for example specialization on benthic invertebrates, aquatic plants, and zooplankton (Uchii et al., 2006). Thus, metagenomics integrates information on commonly consumed prey species and the intestinal microbiota. Furthermore, metazoan prey remnants may be more readily identifiable via sequencing than taxonomic identification of highly digested remains (Leray et al., 2012, 2013, 2015).

Exploring the utility and feasibility of this approach, we considered hawkfish in a *Paracirrhites* species complex from the remote island of Flint (Republic of Kiribati), in the likely center of the distribution for this genus in the Polynesian province of the Pacific (Donaldson, 1988). Hawkfish, including those in the genus *Paracirrhites*, are harvested for the aquarium trade (Donaldson, 2002), and rare species and colorations are highly prized. The arc‐eye *Paracirrhites* group has been identified as a particularly interesting species complex for studies of biogeography (Donaldson, 1986). Donaldson (1986) noted the existence of two centers of distribution for Cirrhitidae, based on high levels of endemism. The center of maximum diversity for *Paracirrhites* in the arc‐eye complex conflicts with a traditional view of Indo‐West Pacific reef fish zoogeography in which there is a single center of highest diversity, the Indo–Malay–Philippine (IMP) biodiversity hotspot (Ekman, 1953), and instead points toward a hypothesis (Woodland, 1983, Gaither & Rocha, 2013) in which high levels of species richness in the IMP are due to pooling of species numbers from two distinct centers of distribution in confluence (center of overlap). In support of this, of the six Indo‐West Pacific species of *Paracirrhites*, all six occur in the Polynesian area; three (*P. nisus*, *P. xanthus*, and *P. bicolor*) are endemic (Donaldson, 1988).

Collection of species in their center of maximum diversity and endemism in the Polynesia province, along with the use of next‐generation sequencing, enabled us to simultaneously re‐examine phylogenetic relationships on the basis of new genetic information and assess the potential role for trophic partitioning in maintaining coexistence among closely related and morphologically similar species. For this study, we hypothesized that species of the arc‐eye complex would exhibit distinct intestinal microbiota and differences in prey remnants obtained from guts, as a partial explanation for potential coexistence of this enigmatic species complex at the center of these species’ ranges. We further hypothesized that the three species endemic to the Polynesian province (*P. bicolor*, *P. xanthus*, and *P. nisus*) would show distinct gut microbiota and prey profiles from the more broadly distributed *P. arcatus*, thus exhibiting a relationship between dietary patterns and biogeographic distribution.

## 2 METHODS

### 2.1 Study system

**Figure 1:**
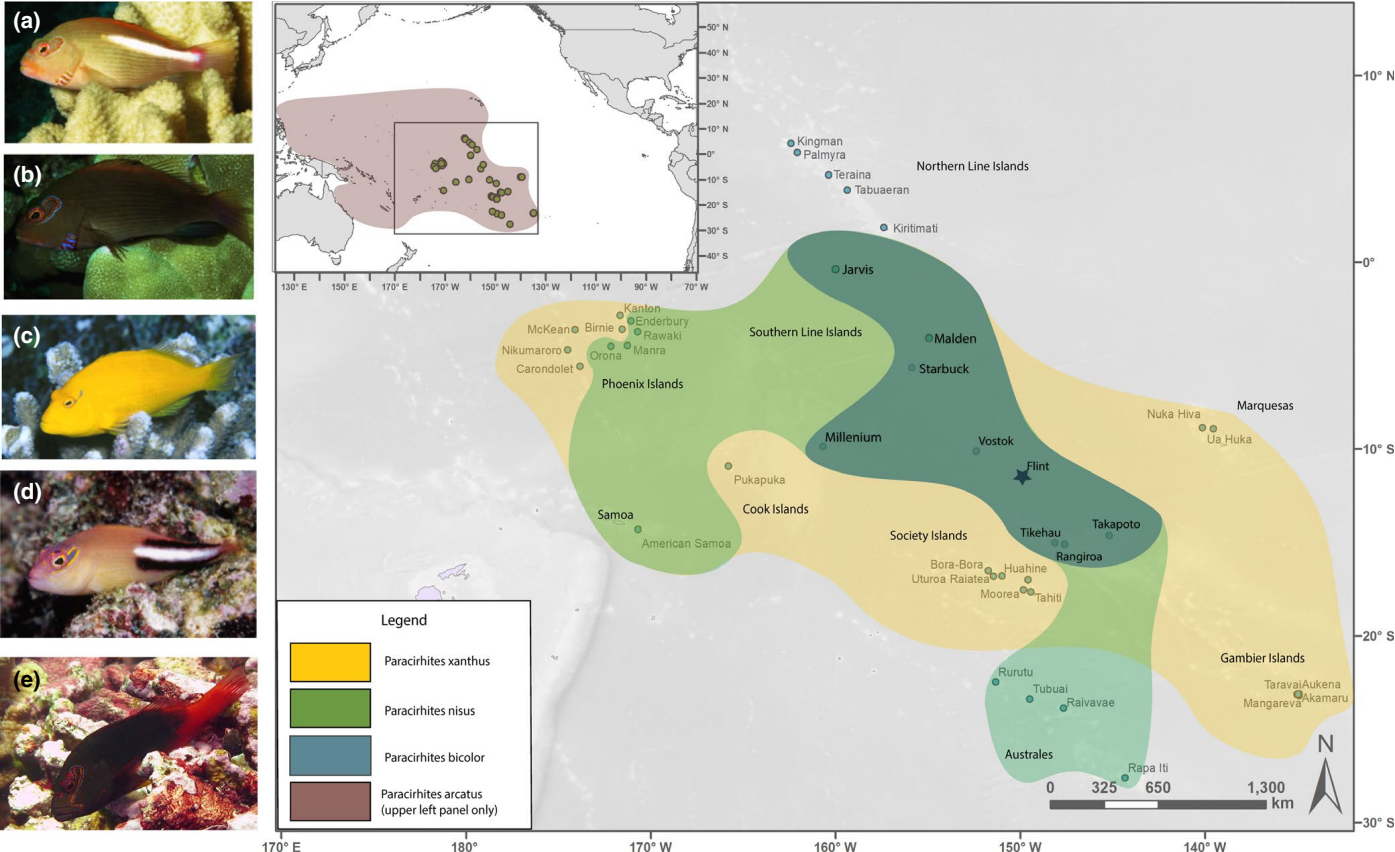
(*Left panel*) Illustration of color and morphological differentiation for species in the *arc‐eye* species complex. Panel (a) corresponds to *Paracirrhites arcatus*; light morph; panel (b) corresponds to *Paracirrhites arcatus*, dark morph; panel (c) is *Paracirrhites xanthus*; panel (d) is *Paracirrhites nisus*; and panel (e) is *Paracirrhites bicolor.* (*Right panel*) Biogeographic range for species in the *arc‐eye* complex. The range of *Paracirrhites arcatus* is shown in the upper left (smaller figure) only, as this range encompasses the ranges of the other species. The square box denotes the enlarged portion. The sampling site for collection of specimens for metagenomic analysis is labeled with a star (Flint Island, Republic of Kiribati). (Photographs: B.J. Zgliczynski and M.J. Adams)

*Paracirrhites arcatus* species complex: Hawkfish in the genus *Paracirrhites* are small (maximum published length 29 cm) coral reef fish in the family Cirrhitidae found across the Indo‐Pacific (Randall, 1963; Randall, Allen, & Steene, 1990). These fish are sit‐and‐wait mesopredators lacking a swim bladder, generally found perched on branching coral species, such as *Pocillopora* (DeMartini, 1996). Morphological analysis of prey remains in gut contents from *P. arcatus* indicate a general diet of small fishes, shrimps, crabs, and other crustaceans (Randall et al., 1990), and a recent DNA metabarcoding approach revealed unexpected prey items such as *Paracalanus parvus*, a pelagic copepod species, in addition to coral crabs (genus *Trapezia*), species in the genus *Galathea* (squat lobster), and traces of DNA of *Pocillopora* coral (Leray et al., 2013, 2015). Although there are six recognized species in the genus *Paracirrhites*, there are only four recognized species of *Paracirrhites* with a characteristic and colorful oblique U‐shaped mark behind the eye; throughout the rest of this manuscript, we will refer to this group as the “arc‐eye complex.” Of the four species in the arc‐eye complex, one (*P. bicolor*) is listed as data deficient by the IUCN, and remaining three as least concern (*P. arcatus, P. nisus, and P. xanthus*). *P. arcatus* is one of the more abundant and widespread of the cirrhitids, known from east Africa to Polynesia. This species occurs in two different color morphs: a light grayish brown to reddish body with a pale pink to white band over the region of the lateral line, and a variety that is olive to dark brown and lacks the pale band on the body. These color morphs appear to be behaviorally unmodifiable, and are unrelated to ontogeny, sex, body size, or maturation (DeMartini & Donaldson, 1996; Donaldson, 1990; Myers, 1989; Randall, 1963; Sadovy & Donaldson, 1995), but differences in microhabitat have been described (DeMartini & Donaldson, 1996; Whitney, Donahue, & Karl, 2018), and they have been considered a potential instance of incipient speciation (Whitney, Bowen, & Karl, 2018). Across the Pacific where the species spans a depth range of 1–27 m, the light color morph was found to be more abundant at depths greater than 10 m (DeMartini & Donaldson, 1996). A more recent examination of microhabitat associations in Hawaii found that the light color morph was observed more frequently in deeper, subsurge zones, the dark morph was found more frequently in shallow, steep surge zones, and phenotypic intermediates were restricted to intermediate habitats (Whitney, Donahue, et al., 2018). The distribution and ecology of the endemic species (*P. xanthus*, *P. nisus*, and *P. bicolor*) are understudied and less understood than for *P. arcatus*. Representative photographs of each species and color morph, as well as distributions from occurrence records, are shown in Figure 1.

### 2.2 Range determination and implications for biogeography

Occurrences of the four species were tabulated based on review of the literature, museum records, and observations from belt‐transect surveys (see Table S3 and S4). Most surveys did not distinguish between color morphs of *P. arcatus,* so we have shown the range as one group in Figure 1. Some reports included relatively broad ranges (island groups; for example, Gambier, Society Islands, Tuamotus) and so we also included shaded ranges encompassing the entire region (eg., Siu et al., 2017), as detailed species lists were not always available by individual island in these areas. Observations of a presumed hybrid (e.g., *P. xanthus* by *P. nisus*) in transect surveys are shown on a map in Figure S2, in the context of observations of *P. xanthus*, *P. nisus,* and *P. bicolor.* Observations of *P. nisus* via visual census have also been recorded in Maratua Island, Indonesia (Madduppa, 2012). It is possible that these observations of *P. nisus* are misidentifications of *P. arcatus,* as *P. nisus* is considered endemic to the eastern central Pacific, and there are no voucher specimens or photographs to provide a definitive identification.

### 2.3 Data collection

Samples were collected from Flint island (11.43˚S, 149.82˚W) in the Southern Line Islands (central Pacific) in the Republic of Kiribati. Sampling included five adult individuals of each of the five groups: *P. arcatus* (light morph), *P. arcatus* (dark morph), *P. bicolor*, *P. nisus*, and *P. xanthus*. Sampling for species was conducted from October 17th‐21st, 2013 and restricted to forereef habitat at an average depth of 10 m to minimize variation due to sampling. (We will refer to these five groups operationally as “species” for brevity. Although the two color morphs of *P. arcatus* are not presumed to be taxonomically distinct, Whitney, Bowen, et al., 2018 found evidence for partial reproductive isolation and potential incipient speciation.) Individual fish were collected with 3‐prong spears (Hawaiian slings) and fish anesthetic (clove oil). Upon collection, fish were stored on ice and brought back to the research ship for initial processing. Each fish was assigned a unique identification tag, and basic morphometric information was collected. Intestines were dissected, and hindgut homogenates from the five individuals per species were combined for DNA extraction using the MO‐BIO Powersoil^®^ DNA Isolation Kit, following the manufacturer's instructions. Sequence libraries were prepared using the Ion XpressTM Plus Fragment Library Kit (Life Technologies, NY, USA) with slight protocol modification, and each library was barcoded using the Ion XpressTM Barcode Adaptors 1–16 Kit. SPRI beads‐based size selection was performed according to the published New England Bioscience (NEB) E6270 protocol (https://www.neb.com/protocols/1/01/01/size-selection-e6270) for 200–300 bp fragment size selection after adaptor ligation. Emulsion PCR was performed on an 8‐cycle amplified library using the OneTouch supplemented with Ion Torrent PGM Template OT2 200 Kit. Template libraries were sequenced on the Ion Torrent PGM using the Ion Torrent PGM Sequencing 200 Kit v2 and Ion 318TM Chip Kit v2 on the research vessel M/Y Hanse Explorer (Lim et al., 2014).

### 2.4 Bioinformatic analyses

DNA isolated from gut homogenates was pooled for five individuals per species and sequenced to yield 5 metagenomic libraries (*P. arcatus*: light morph, *P. arcatus*: dark morph, *P. xanthus*, *P. nisus*, and *P. bicolor*). Metagenomic sequence reads were filtered for quality using the Preprocessing and Information of Sequences tool (PRINSEQ; Schmieder & Edwards, 2011). The sequenced reads were translated in silico into predicted protein sequences and compared with the SEED database to provide taxonomic identifications and information on organism abundance using Blastx (Atschul, Gish, Miller, Myers, & Lipman, 1990) with a maximum e‐value cutoff of 1 × 10^‐5^, a minimum alignment cutoff of 15, and a minimum percent identity of 80%. Metagenomes were deposited in the MG‐RAST metagenomics analysis server (Meyer et al., 2008) and made publicly available using the unique identifiers listed in Table S1. Taxonomic assignment of bacterial species was confirmed using a blastn search against whole bacteria genomes deposited in GenBank (ftp://ftp.ncbi.nlm.nih.gov/genomes/Bacteria/). Bacterial taxa were categorized at the phylum level, with the exception of Proteobacteria and Firmicutes, which were categorized by class. Unclassified bacteria were categorized as “other bacteria.” While conducting analyses at this taxonomic level aids in comparability to results from other studies conducted on fish gut microbiome, there may be hidden diversity in the identity and function of the gut microbial community that warrants a deeper look. For this reason, the 16s rRNA, rpoB, and recA gene sequences were extracted from the unassembled reads of each genome and clustered into OTUs using the program GenomePeek (McNair & Edwards, 2015). Further analyses focused on the results for 16S rRNA exclusively, as the database for this marker has the most coverage, thereby making comparisons of relative sequence abundance more accurate. For the analysis of putative prey items, the metagenomes were subset into 20,000 sequences to speed processing time. We used Midori‐UNIQUE, a curated reference dataset of metazoan mitochondrial DNA sequences containing all unique haplotypes for each species for 16,697 unique species (Machida, Leray, Ho, & Knowlton, 2019) as our reference dataset. The Midori reference datasets are divided into multiple markers, including two ribosomal RNA and thirteen mitochondrial protein‐coding genes found in most phyla of metazoans. The cytochrome oxidase subunit I (COI) gene had the largest number of sequences, so we conducted our analyses with this dataset. We performed an initial blastn search for each metagenome in CLC Genomics Workbench (Qiagen Bioinformatics). Retaining only those sequences with a maximum e‐value cutoff of 1 × 10^‐4^, we then removed sequences with matches to *Paracirrhites*, as we assumed these were likely the result of host tissue contamination, rather than cannibalism, as customary for these analyses (Casey et al., 2019). We took all remaining annotations and calculated relative abundance within each metagenome at the phylum level. At lower taxonomic levels (eg. species), the best sequence hit sometimes matched to an organism that either (a) does not occur in the study location, according to existing censuses, or (b) does not occur in the marine environment (e.g., species in the orders Lepidoptera and Hemiptera within the phylum Arthropoda). To avoid any erroneous assumptions produced by using false species IDs or excluding important prey that could not be confidently assigned to lower taxonomic levels, we therefore conducted all analyses with metazoan sequences at the phylum level.

### 2.5 Phylogenetic analyses

Sequence reads from the metagenome for each species and color morph were aligned against the reference genome for the *P. arcatus* mitochondrion (17,336 bp, NCBI RefSeq AP006012.1) using alignment tools in CLC Genomics Workbench 7 (Qiagen, CA, USA). Using *Cheilodactylus quadricornis* (NCBI RefSeq: KT357695.1), a species in the sister group to Cirrhitidae (Donaldson, 1997), and *Cirrhitichthys aprinus* (NCBI RefSeq: AP006011.1) as outgroups, the resultant FASTA mitochondrial DNA (mtDNA) sequences were aligned using MAFFT, a multiple alignment program for amino acid or nucleotide sequences. Maximum likelihood (ML), maximum parsimony (MP), and Bayesian inference (BI) analyses were conducted in order to assess congruence between the methods. An ML tree was constructed with RaxML‐HPC2 (Randomized Axelerated Maximum Likelihood) using the GTRGAMMA model with 100 bootstrap replications (Stamatakis, 2014) run on the CIPRES portal (Miller, Pfeiffer, & Schwartz, 2010). MP was carried out using PAUP 4.0a166 (Swofford, 2003). Node support was assessed using jackknifing of sites with 100 replicates to produce a 50% majority‐rule consensus tree (Figure S3). BI was conducted in MrBayes v.3.2.7a (Ronquist & Huelsenbeck, 2003) using two runs of four chains with ten million generations and a sampling frequency of 1,000 generations for the Markov chain Monte Carlo analysis. The first 25% of trees were discarded as burn‐in and the posterior probabilities were estimated by combining the remaining trees from each run into a majority‐rule consensus tree.

### 2.6 Statistical analyses

In order to assess differences in bacterial community composition and metazoan prey composition between the four species and two color morphs, separate similarity profile routine (SIMPROF) tests were conducted on relative abundance of bacterial taxonomic groups and the relative abundance of metazoan sequences using the *clustsig* package (Whitaker & Christman, 2010) implemented in R (R Development Core Team, http://www.r-project.org). SIMPROF is a permutation procedure, which tests for the presence of sample groups in a priori unstructured sets of samples (Clarke, Somerfield, & Gorley, 2008). Analyses were based on 10,000 random permutations of the annotated metagenomic data. Following procedure which has been used in other analyses of bacterial structure in metagenomic samples, the two most abundant phyla (Firmicutes and Proteobacteria) were further divided into the lower taxonomic grouping of class to provide clearer resolution of the groups driving differences in microbiome composition between species (Kelly et al., 2014). Shannon–Weiner (H′) diversity indices were calculated using the R package *vegan* (Oksanen, Blanchet, & Kindt, 2012). All statistics were conducted using R 3.1.2.

## 3 RESULTS

### 3.1 Phylogenetic analyses of hawkfish

The ML and MP phylogenetic analyses of the whole mitochondrial genome (mtDNA) for all four species, including the two color morphs analyzed separately, provided almost identical reconstructions with strong node support for the separation of two clades within the arcatus complex, with one clade composed of *P. arcatus* (both color morphs) and *P. bicolor*, and one clade composed of *P. xanthus* and *P. nisus*. The maximum likelihood tree showed clustering of *P. xanthus* with *P. nisus*, in addition to clustering of the two color morphs of *P. arcatus* with *P. bicolor.* Under maximum parsimony and Bayesian inference, the phylogenetic tree showed additional structure among species, with *P. arcatus* (dark morph) and *P. bicolor* forming a sister clade to *P. arcatus* (light morph) (Figure 3, *right*).

### 3.2 Fish gut microbiomes and metabolic functions

**Figure 2:**
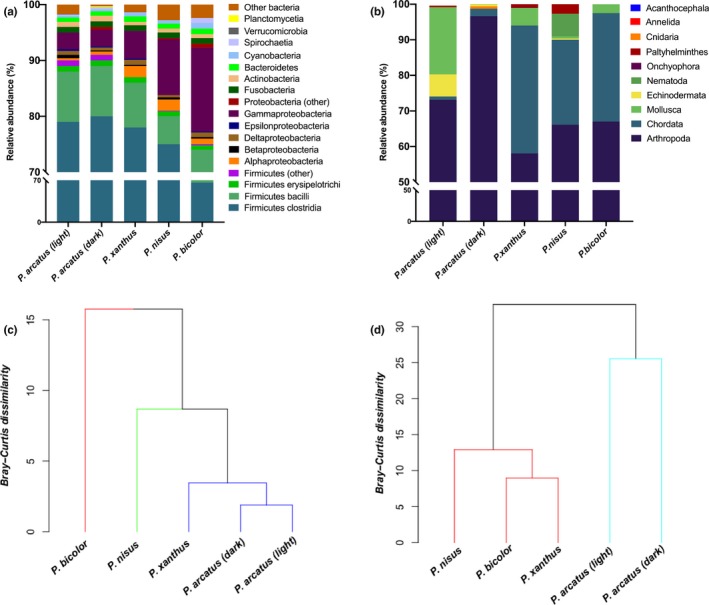
(a) *Gut microbial community structure and metazoan composition of metagenomes*. Stacked barplot illustrating relative abundance of bacterial phyla within total bacterial reads. The two most abundant phyla (Proteobacteria and Firmicutes) are broken down into class. (b) *Putative prey sequences in hawkfish gut contents*. Stacked barplot illustrating relative abundance of all High‐scoring Segment Pairs (HSPs) with e < 1 × 10^‐4^ from BLASTn search of the Midori‐UNIQUE reference dataset for the cytochrome oxidase subunit I (COI) gene, after removal of HSPs where the accession was a match to *Paracirrhites*. (c) Dendrogram from Similarity Profile Routine (SIMPROF) test on hawkfish gut microbial community structure. Colors indicate significantly different clusters at α = .05. (d) Dendrogram from Similarity Profile Routine (SIMPROF) test on metazoan prey sequence composition in hawkfish guts. Colors indicate significantly different clusters at α = .15

**Figure 3:**
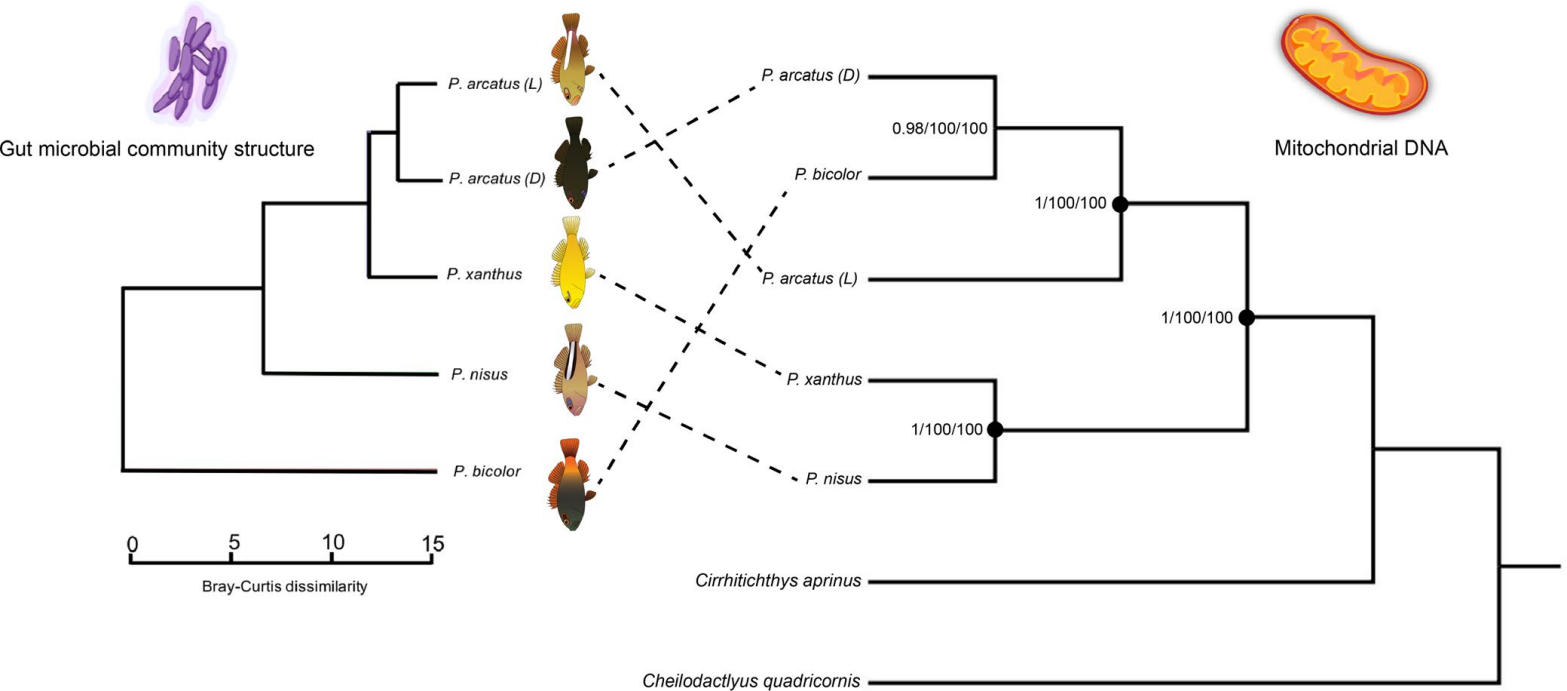
Dendrogram from SIMPROF analysis on hawkfish gut microbial communities (left). *Paracirrhites bicolor* and *Paracirrhites nisus* show significant (*p* < .05) separation from *Paracirrhites arcatus* (both color morphs) and *Paracirrhites xanthus*. The microbiomes of the two color morphs and *Paracirrhites xanthus* are not significantly different from one another. Phylogenetic analysis of hawkfish species using genome sequences for the mitochondrion (right). Depicted is a phylogenetic tree for *Paracirrhites* species with *Cheilodactlyus quadricornis* (KT357695.1) and *Cirrhitichthys aprinus (*AP006011.1) as outgroups. Support values are indicated at the nodes in the order Bayesian inference/maximum likelihood/maximum parsimony. Maximum support values (1/100/100) are indicated at the nodes by black circles

Investigation of the bacterial community structure shows divergence between species, but not color morphs (SIMPROF, α < .05, see Figure 2). Firmicutes and Proteobacteria together made up 94%–96% (Firmicutes = 81%–91%; Proteobacteria = 5%–13%) of the bacterial community and thus were divided into class. SIMPROF analysis of bacterial community structure in the five composite metagenomes for each of the five members of the arc‐eye complex revealed three significant (α = .05) groupings: (1) *P.arcatus* (light), *P.arcatus* (dark), and *P. xanthus*; (2) *P. nisus*, and (3) *P. bicolor* (Figures 2c and 3). From the analyses of 16S rRNA OTUs, *Clostridium perfringens* was found in highest abundance of all bacterial taxa for all species, but comprises only 42.6% and 37.4% of the relative abundance of all 16S contigs for *P. nisus* and *P. bicolor*, respectively, in comparison with 78%–94% of the bacterial community for *P. xanthus* and *P. arcatus* (both morphs). Accordingly, Shannon–Weiner diversity indices of the gut microbial communities using 16S data are highest for *P. bicolor* (H′ = 2.27) *and P. nisus* (H′ = 1.51)*,* and consistent with the clustering identified at higher taxonomic levels (Figure 2c).

### 3.3 Putative prey

Chordate sequences were found in higher relative abundance for the species with a more restricted range (*P. nisus*, *P. xanthus, and P. bicolor*); that is, 24%–36% compared to 0.9%–2% for *P. arcatus* (dark) and *P. arcatus* (light), respectively (Figure 2b). SIMPROF analysis of the metazoan data (α = .15) shows two groupings; grouping of the two color morphs of *P. arcatus,* and a separate grouping with the three endemic species (*P. bicolor*, *P. xanthus*, and *P. nisus*), see Figure 2d.

One limitation of this approach is the difficulty in distinguishing secondary predation (that is, prey remains in a prey item's gut) from targeted prey, or incidental consumption in the course of feeding. For instance, sequences with a best match to Cnidaria were found in the metagenome for the dark morph of *P. arcatus*. *P. arcatus* are known to feed on mutualistic coral crab species of the genus *Trapezia* (Leray et al., 2015), which themselves feed on coral mucus (Knudsen, 1967). Given the low relative abundance of sequences matching to Cnidaria (>1%), it is certainly possible these sequences are from secondary prey.

## 4 DISCUSSION

The importance of the expansion of feeding forms over evolutionary time for the diversification of reef fishes has been well‐supported, with niche partitioning and morphological innovation identified as playing key roles in the development of herbivory and detritivory (Bellwood et al., 2014; Bellwood & Wainwright, 2006). Behavioral innovations may be equally as important as morphological innovations but have not received the same attention likely due to difficulties in inference from the fossil record. Diversification by this mode, however, may have left traces in the current biogeography of reef fishes, and may be more readily studied by examining potential ecological differentiation in present‐day genera. Local adaptation may reduce the likelihood of successful colonization of novel habitats after dispersal, and dietary specialization has been linked to reduced species durations (Balisi, Casey, & Van Valkenburgh, 2018), as many of the traits that increase the chance of speciation also increase the chances of extinction (Jablonski, 2008). Compressed ranges may therefore be the result of either (a) limitations in dispersal or successful colonization, or (b) the result of past extinction events. Simultaneous examination of phylogenetic relationships, potential ecological differentiation, and current ranges within a genus—especially one for which incipient speciation has been proposed (Whitney, Bowen, et al., 2018)—may provide clues to the mechanisms involved in creating and maintaining the observed species’ distributions. To that end, our findings point toward previously unidentified relationships in a cryptic species complex at its proposed center of distribution, and discordance between gut microbial communities and phylogeny of the host fish which support the hypothesis of niche separation.

The findings here further demonstrate that metagenomic techniques can provide data comprising three important lines of information for a variety of uses: (1) host genetics, (2) microbial communities in the gut, and (3) the identity of putative prey items. When these tools were applied to understudied and closely related fish from a remote island, the value of the multidimensional data are revealed well. Species‐level designations for hawkfish included in this study were originally determined on the basis of morphometric differences. In the initial description by Randall (1963), it was suggested that, of the five species with postocular marks in *Paracirrhites*, *P. bicolor* might be most closely related to *P. nisus*. Our phylogenetic analysis of mtDNA, in contrast, suggests that *P. nisus* and *P. xanthus* are more closely related than *P. bicolor* and *P. nisus*. This finding further matches observations of *P. nisus* by *P. xanthus* hybrids observed in the wild (see Figure S1, Tables S3 and S4), as species that are more closely related are more likely to hybridize. The phylogenetic analyses conducted here further raise the possibility that *P. bicolor* is a color morph variation of *P. arcatus*, given that mtDNA sequences for the *P. arcatus* dark morph and *P. bicolor* were nearly identical (>99% similarity). As mtDNA is inherited solely from the female parent, it is also possible that *P. bicolor* is a hybrid arising from a spawning event in which *P. arcatus* was the female parent with an unknown male species. In support of this possibility is the extreme rarity of *P. bicolor*. Rarity of at least one parental species has been shown to play an important role in hybrid formation for some reef fishes, with hybridization resulting from mating of closely related species due to scarcity of conspecific partners (Hobbs, Frisch, Allen, & Herwerden, 2008). Incorporating nuclear markers with the mtDNA results, as well as microsatellite markers, would further clarify phylogenetic relationships between species in this study. These possibilities are being investigated with additional molecular research.

A unique gut microbiome and differential relative abundance of likely dietary items suggests that there may be some ecological differences among species, despite high genetic similarity, that warrant further investigation. Previous studies have demonstrated an unexpected negative relationship between diet diversity and diversity of gut microbiota for two fish species; that is, dietary generalists had a less diverse microbiome (Bolnick et al., 2014). Indeed, we found that *P. bicolor* and *P. nisus* have the most diverse microbiota, perhaps indicating dietary specialization. Additionally, although the two color morphs of *P. arcatus* cluster in analyses of gut microbial communities and metazoan sequences, differences in relative abundance of sequences for prey items found in the guts warrant further study of potential fine‐scale differences. As noted previously, it has been suggested that *P. arcatus* may be an example of incipient speciation in a sympatric species (Whitney, Bowen, et al., 2018). Differences in composition of diet between color morphs could be driven by these differences in microhabitat associations.

Analyses of putative prey remains revealed broad convergence with what was generally known regarding *Paracirrhites* diets, including a high relative percentage of sequences belonging to the phyla Arthropoda and Chordata. This is consistent with a reported diet consisting predominantly of crustaceans and other fishes, but also revealed some interesting differences. In particular, the three species with the most restricted ranges had a higher relative abundance of sequences matching Chordata. An analysis of gut contents by morphologic identification of prey remains for 199 individuals of *Paracirrhites arcatus* (both color morphs) show a range of 0.62%–9.4% relative abundance for other fish species (Cordner, 2013, see Table S2), suggesting that other fish species may make up a higher proportion of the diet for those species with more restricted ranges (*P. bicolor*, *P. nisus*, and *P. xanthus*). One potential caveat is that all sequences with a match to the host organism were removed to avoid conflation of host tissue with estimates of prey consumption. Any instances of intraspecific predation would therefore be impossible to detect. As this processing was applied equally across all species, if intraspecific predation is more frequent among *P. arcatus*, for instance, the estimates of relative abundance of prey fish would be underestimated in comparison.

There are some considerations that must be noted for the interpretation of our results, and in particular what the relative abundance of metazoan sequences indicates for hawkfish diets over long timescales. The number of High‐scoring Segment Pairs (HSPs) is not a direct correlate to the number of organisms in the gut; a single large organism could result in many HSPs, and gene copy number differs among eukaryotes (Prokopowich, Gregory, & Crease, 2003). Furthermore, carnivorous fish have relatively short gut passage times (~8 hr, Clements, Angert, Montgomery, & Choat, 2014), so the identification of prey remains, even with combined samples to homogenize variability, only reflects a limited selection of what these fish are eating over time. Gut microbiomes may therefore be a more stable predictor of integrated food habits over time. Dietary interventions in well‐studied human gut microbiomes have shown community changes on timescales of months (Ley, Turnbaugh, Klein, & Gordon, 2014) to years (Faith et al., 2013), and others showing return to original structure of gut microbiota only 2 days after the end of a dietary intervention (David et al., 2014). Studies that have examined this for fish have had similarly varying results. A study on captive clownfish found that certain bacterial groups such as *Clostridia perfringens* did not vary significantly with time postfeeding, whereas other groups such as *Bacilli* (Firmicutes) and *Photobacterium sp.* increased after feeding (Parris, Morgan, & Stewart, 2019). Other studies found no or minimal changes in diversity, but observed changes in relative abundance of bacterial phyla 3–24 hr after feeding (Zhang et al., 2018) and 3‐12 days post‐fasting (Xia et al., 2014). Taken together, there does appear to be a core microbiome for fish that is sensitive to diet on longer timescales. We further posit that the general concordance between the metazoan and microbial data that we show here provides strong evidence for real ecological differences between species.

Some of the limitations associated with this method can help guide additional research in this field and contribute to the discussion on best practices in using next‐generation sequencing of gut contents as a technique for assessing dietary habits within animals. As guts were pooled to produce composite metagenomes, information on individual variability between microbiomes and diet within species was impossible to obtain for this study, although we had a priori evidence that intraspecific variability in this genus would be low. In analyses excluding all empty stomachs (~10%), stomach content analysis of 199 *P. arcatus* individuals was indistinguishable across islands (Zgliczynski et al. 2019). In instances where there are constraints to the number of samples that can be analyzed, whether financial or logistical, specimen pooling is often promoted as an option with very little loss of statistical power (Weinberg & Umbach, 1999). Shisterman and Vexler (2008) show that estimations based on pooled data increase efficiency over the use of individual measurements when an assay has a detection threshold, as pooling samples minimize the amount of information lost below the detection threshold. This technique is used extensively in the literature for population‐based epidemiological studies (Keeler & Berwick, 1976; Shisterman & Vexler, 2008) and other situations with high‐dimensional biological data, such as that generated via high‐throughput sequencing techniques (Clarke, Ressom, et al., 2008).

Sample sizes for this study were also limited. *P. bicolor* in particular is so rare that a photograph of one was only published recently (Bacchet, Zysman, & Lefevre, 2017), and this species is known from only four specimens from two localities (Randall, 1963). Future studies that incorporate analyses of intraspecific variability with higher sample sizes for species where additional collection is both logistically possible and ethically defensible are warranted to verify and extend the findings here.

Notwithstanding the limitations, a major strength of this approach includes the ability to look at the host genetics, microbiome, and the presumed prey fragments simultaneously. This approach further allows the investigation of the results using multiple genes, rather than one preselected marker that must be chosen a priori (as in the use of metabarcoding). In most situations, and particularly for highly complex ecosystems, reference barcodes are not available for all potential prey species. Combined with the knowledge that no single marker is ideal for resolving all taxonomic groups (Deagle, Jarman, Coissac, Pompanon, & Taberlet, 2014; Hebert, Ratnasingham, & deWaard, 2003), the use of this technique represents a significant advancement in the available tools for investigating realistic patterns of prey consumption in wild animals. As databases further improve through international efforts, our ability to address complex ecological questions with genetic tools will continue to grow. It is our hope that this study will encourage more community ecologists and conservation scientists to consider metagenomics as a powerful tool that can be used to leverage multidimensional and ecologically informative data from rare and understudied species.

## CONFLICT OF INTEREST

None Declared.

## AUTHOR CONTRIBUTIONS

BZ and SS initiated the project and collected the samples. YL conducted the sequencing on the expedition. BF conducted the analyses and wrote the paper. RE, YL, and FR contributed to the analyses and design thereof. All authors contributed to the final version of the manuscript.

## Supporting information

Supinfo

TableS3

TableS4

## Data Availability

Metagenomes were deposited in the MG‐RAST metagenomics analysis server (Meyer et al., 2008) and made publicly available using the following unique identifiers: *P. arcatus*; light morph: mgm4632779.3, *P. arcatus*; dark morph: mgm4856250.3, *P. xanthus*: mgm4632786.3, *P. nisus*: mgm4631833.3, and *P. bicolor*: mgm4856249.3/mgm4856251.3.
