# Supplementary material for: Decoding diversity in a coral reef fish species complex with restricted range using metagenomic sequencing of gut contents
Source: Ecol Evol. 2020 Mar 10;10(7):3413–23. doi: 10.1002/ece3.6138 (PMC7141070; doi:10.1002/ece3.6138)
Supplement: Supplementary file 1 — Supinfo [file ECE3-10-3413-s001.docx]

*Ecology and Evolution*

**Supplementary Material for:**

**Decoding diversity in a coral reef fish species complex with restricted range using metagenomic sequencing of gut contents**

*Authors:* Beverly J. French^1^*, Yan Wei Lim^2^, Brian J. Zgliczynski^1^, Robert A. Edwards^2,^ Forest Rohwer^2^, Stuart A. Sandin^1^

^1^Center for Marine Biodiversity and Conservation, Scripps Institution of Oceanography, University of California, San Diego, 9500 Gilman Dr. MC 0208, La Jolla, CA 9209300208

USA ^2^Department of Biology, San Diego State University, San Diego, CA 92182

**Table of Contents**

1. **Supplementary Methods**
2. **Supplementary Figures**
   1. **Figure S1.** *Presumed* Paracirrhites nisus *by* xanthus *hybrid.*
   2. **Figure S2.** *Map displaying occurrence records of the P. nisus, P. xanthus, P. bicolor, and P. nisus x xanthus hybrids.*
   3. **Figure S3.** *Jackknife 50% majority-rule consensus tree from maximum parsimony phylogenetic analysis.*
   4. **Figure S4.** *Metabolic functions recovered from metagenomes.*
3. **Supplementary Tables**
   1. **Table S1.** *Details of metagenomic libraries*
   2. **Table S2.** *Visual identification of prey items from gut contents*
   3. **Table S3.** *Transect records of all species in arc-eye complex from available islands (separate attachment)*
   4. **Table S4.** *Information on the literature review and metadata of specimens from museum records.*

**Supplementary Methods**

**Methods for novel and unpublished fish survey records**

For the unpublished survey records, quantitative underwater surveys (belt transect surveys) of reef fish assemblages were conducted, following methodological details described elsewhere (DeMartini et al. 2008, Friedlander et al. 2010, Sandin et al. 2008). We analyzed quantitative survey data collected from islands, atolls, and reefs spanning the equatorial Pacific between 2009-2017. Surveys were restricted to the forereef slope at depths between 8 and 15 m. The unit of replication within islands was the station. Although belt transects can underestimate the presence of cryptic fishes and cryptic species (Willis 2001), it is a non-extractive method that can be used to capture high diversity and species with patchy distributions (Caldwell et al. 2015), and was able to capture all of the species in surveys in the arc-eye complex in their region of maximum diversity in the central Pacific (Line Islands and Tuamotus). Records are provided in Table S4.

**Museum Records**

We used FishNet2, a collaborative effort of global fish collections designed to share and distribute data on specimen holdings from around the world, to search for all records of species in the arc-eye *Paracirrhites* species complex. A total of 723 individual specimen records, and their metadata, were downloaded and are provided in Table S4.

**Parameters for CLC Genomics Workbench BLAST analyses (metazoan sequences):**

Match = 2, mismatch = 3, existence = 5, extension = 2; expectation value = 10; word size = 11; mask lower case = no; filter low complexity = yes; maximum number of hits =20000; number of threads =10. Retaining only those sequences with a maximum e-value cutoff of 1x10^-4^ resulted in 31 annotations for *P. nisus,* 57 annotations for *P. bicolor*, 68 annotations for *P. arcatus* (dark), 61 annotations for *P. arcatus* (light), and 42 annotations for *P. xanthus*.

1. **Supplementary Figures**

**
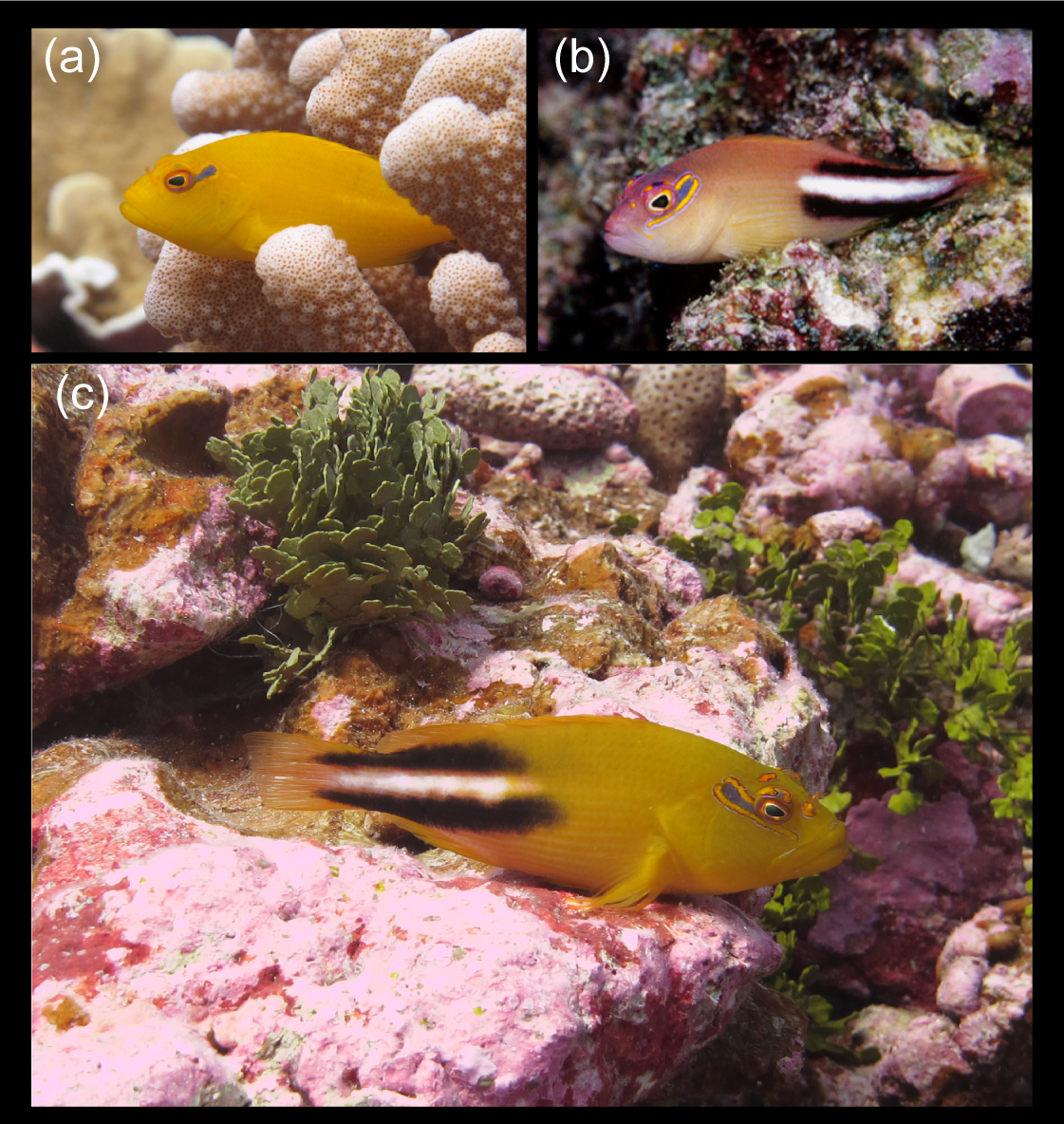
**

**Figure S1.** *Comparison of P. xanthus, P. nisus and apparent P. xanthus x nisus hybrid observed in the Southern Line Islands.* (a) Image of *Paracirrhites xanthus*, photograph taken by Brian Zgliczynski (b) Image of *Paracirrhites nisus*, photograph taken by Mary Jane Adams at Nikumaroro Island (c) Apparent *Paracirrhites xanthus* x *nisus* hybrid observed in the Southern Line Islands, photograph taken by Brian Zgliczynski. *P. xanthus x P. nisus* “hybrids” have been observed in Tikei (Tuamotus, French Polynesia), Millenium Atoll, Vostok, Starbuck, and Flint (Southern Line Islands, Republic of Kiribati).


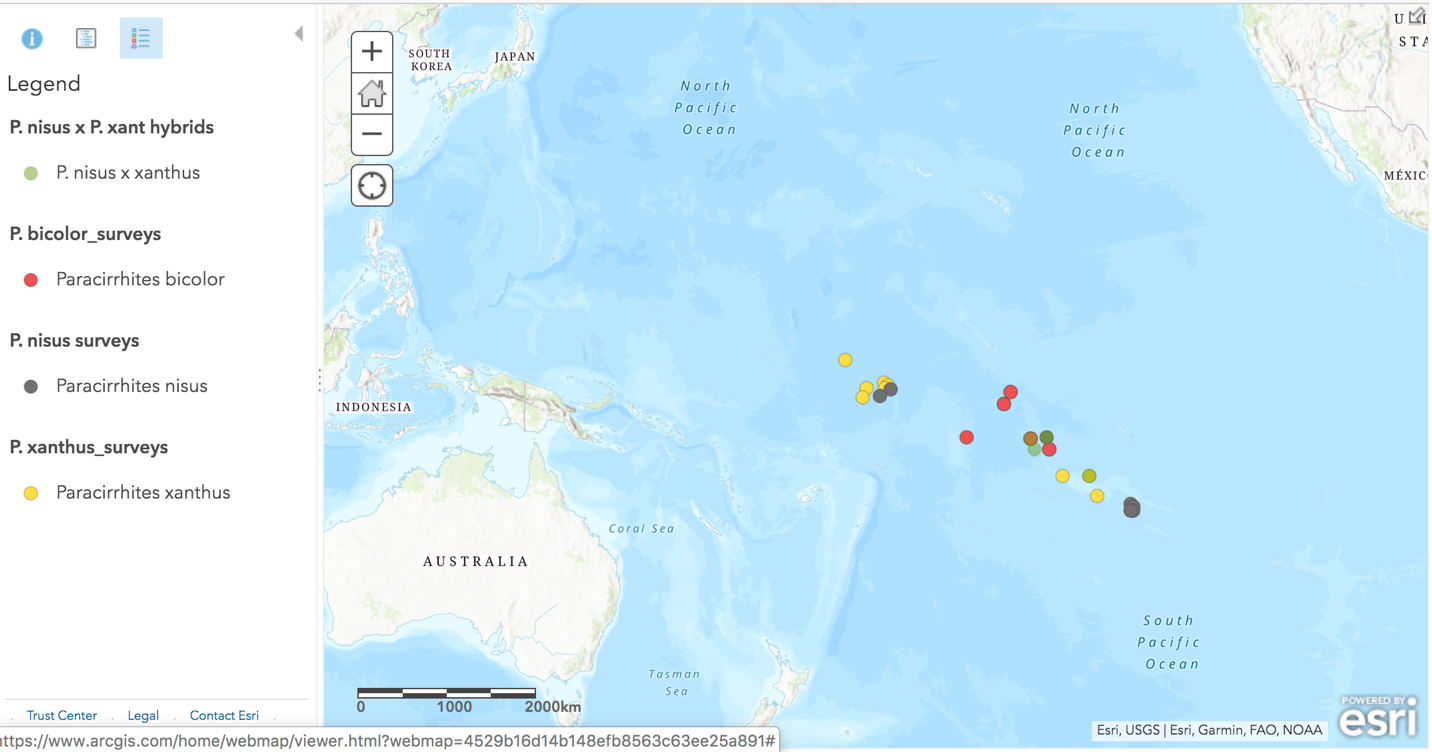

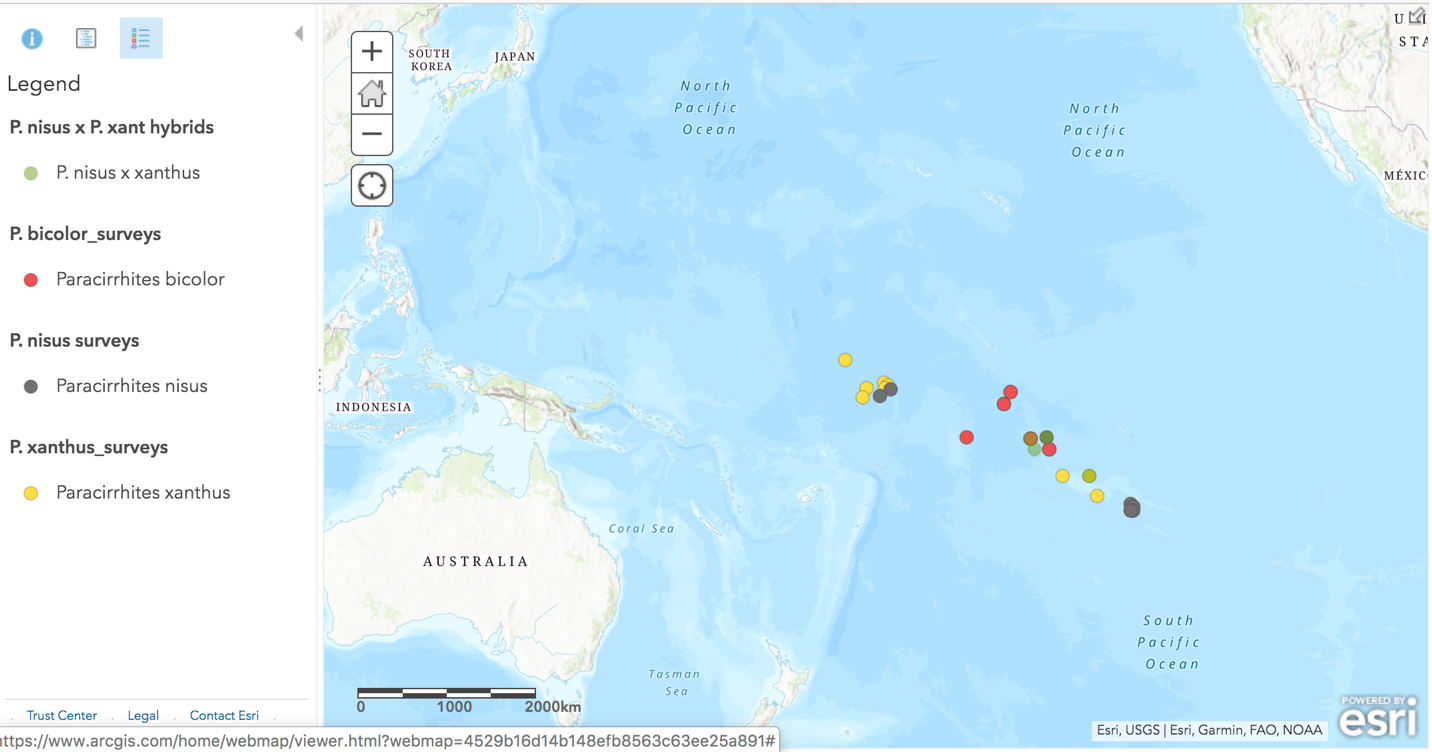


**Figure S2.** *Map displaying occurrence records from visual census surveys of the species considered endemic to the Polynesian province, plus a putative hybrid of P. nisus x xanthus.* Yellow circles correspond to *P. xanthus*, black circles to *P. nisus*, red circles to *P. bicolor*, and green circles to putative *P. nisus x P. xanthus hybrids*. All occurrence records correspond to the survey records included in Table S4.


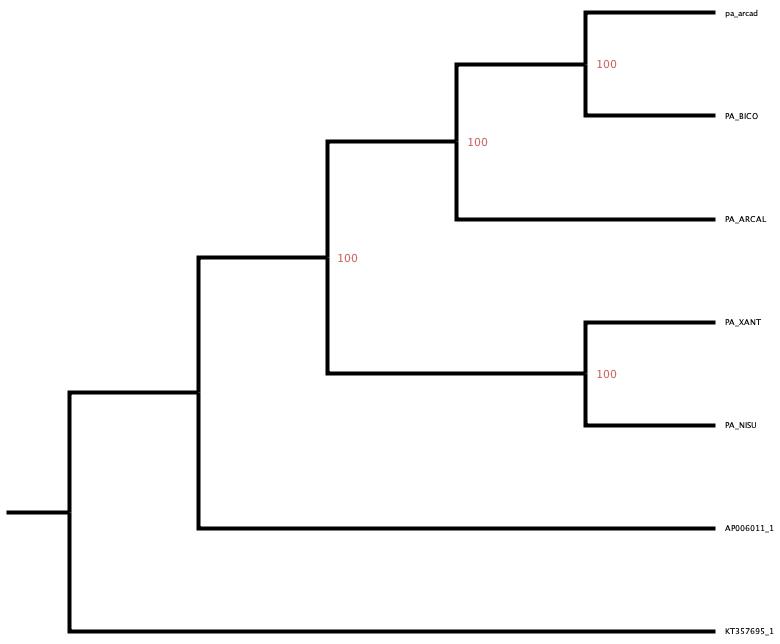


Figure S3. **J*ackknife 50% majority-rule consensus tree from maximum parsimony phylogenetic analysis.*** KT357695.1 is the NCBI GenBank reference for the complete mitochondrial genome for *Cheilodactlyus quadricornis*. AP006011.1 is the NCBI GenBank reference for the complete mitochondrial genome for *Cirrhitichthys aprinus.*

**
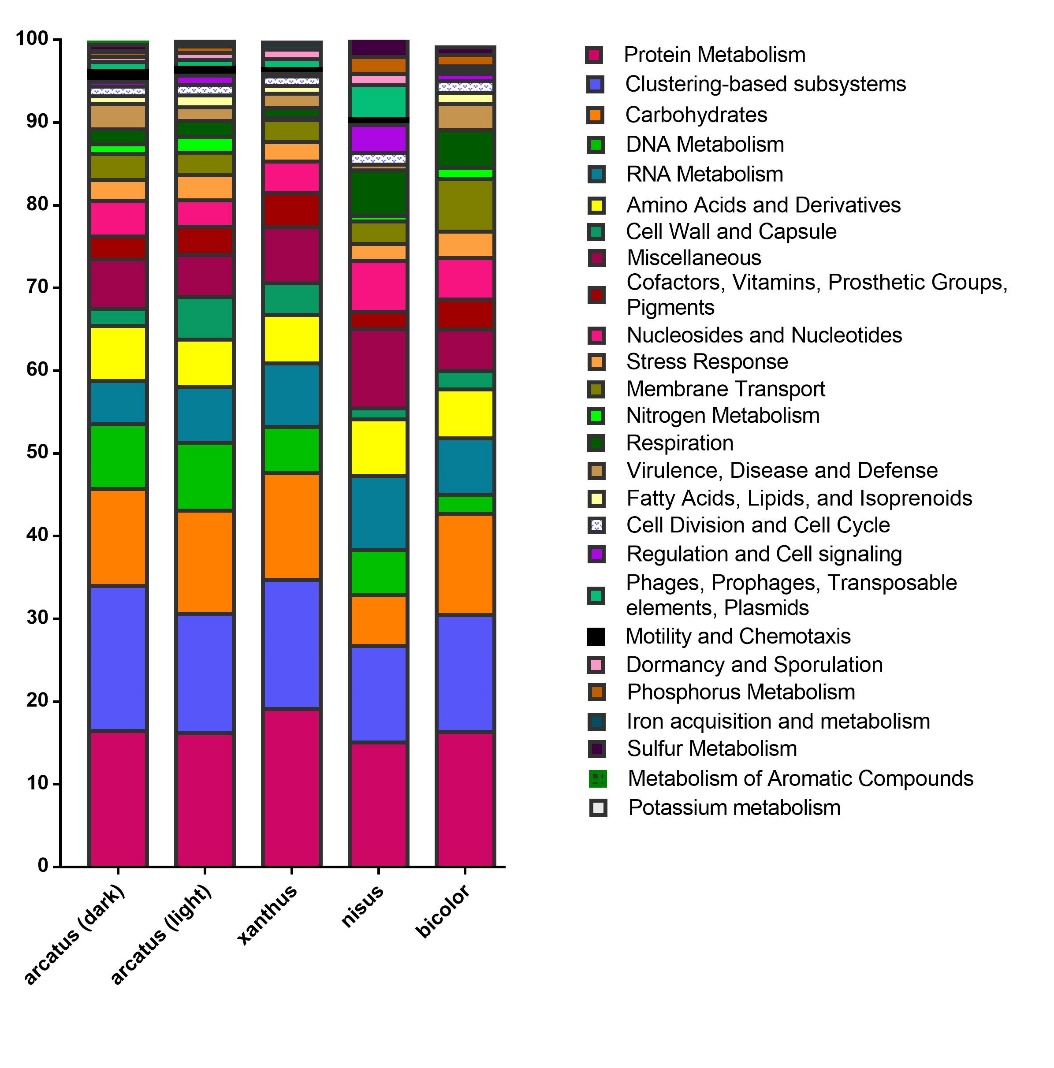
 Figure S3.** *Metabolic functions in metagenomes.* Stacked barplot indicating the relative abundance of genes in functional categories, using level-one SEED subsystem-based functional annotations.

1. **Supplementary Tables**

| **Sample Name** | **MG-RAST IDs** | **Total no. of reads** | **Mean read length (bp)** | **% GC content** |
| --- | --- | --- | --- | --- |
| *Paracirrhites arcatus* (light morph) | mgm4632779.3 | 5,709,803 | 153 ± 56 | 38 ± 10 |
| *Paracirrhites arcatus* (dark morph) | mgm4856250.3 | 1,218,165 | 158 ± 33 | 39 ± 9 |
| *Paracirrhites xanthus* | mgm4632786.3 | 1,277,645 | 151 ± 27 | 41 ± 9 |
| *Paracirrhites nisus* | mgm4631833.3 | 6,223,957 | 152 ± 52 | 42 ± 9 |
| *Paracirrhites bicolor* | mgm4856249.3  mgm4856251.3 | 1,594,441 | 137 ± 46 | 42 ± 9 |

**Table S1.** Details of metagenomic libraries deposited in the MG-RAST server under the study name “SLI_FishGutMetagenomes.”


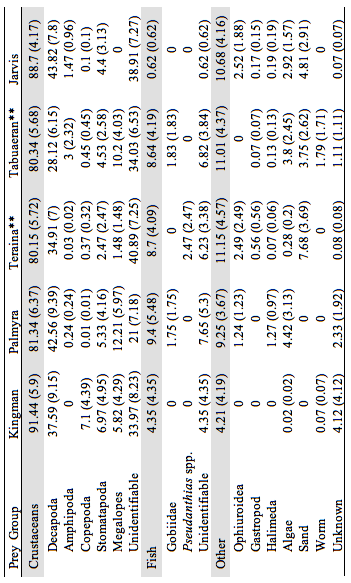


**Table S2.** Mean percentages of prey groups identified in 199 *Paracirrhites arcatus* guts from the Northern Line Islands. Islands listed from North to South (*reproduced from* Cordner 2013). **indicates an inhabited island

**Table S3.** *Transect records of all species in arc-eye complex from available islands (separate attachment).*

**Table S4.** *Information on the literature and museum records, including metadata for the individual publications and specimens (separate attachment).*

**References**

Caldwell, ZR, Zgliczynski BJ, Williams GJ, Sandin SA. (2015) Reef Fish Survey Techniques: Assessing the Potential for Standardizing Methodologies. PLoS ONE 11 (4): e0153066

Cordner, E.G. (2013) Diet Shifts in Coral Reef Fishes in Response to Changes in Food Availability and Predator Density. University of California, San Diego, ProQuest Dissertations Publishing, 2013. 1540928.

DeMartini E.E., Friedlander A.M., Sandin S.A., Sala E. (2008) Differences in fish-assemblage structure between fished and unfished atolls in the northern Line Islands, central Pacific. *Mar Ecol Prog Ser* 365:199-215

Friedlander, A.M., S.A. Sandin, E.E. DeMartini, E.S ala. (2010) Spatial patterns of the structure of reef fish assemblages at a pristine atoll in the central Pacific *Mar. Ecol. Prog. Ser.,* 410, pp. 219-231

Greenfield, D. & Williams, I. 2016. Paracirrhites xanthus. (errata version published in 2017) The IUCN Red List of Threatened Species 2016: e.T67997896A115455316. <http://dx.doi.org/10.2305/IUCN.UK.2016-1.RLTS.T67997896A68001741.en>

Greenfield, D. & Williams, I. 2016. Paracirrhites bicolor. (errata version published in 2017) The IUCN Red List of Threatened Species 2016: e.T67997896A115455316. <http://dx.doi.org/10.2305/IUCN.UK.2016-1.RLTS.T67997896A68001741.en>

Sandin, S.A., Smith JE, DeMartini EE, Dinsdale EA, Donner SD, Friedlander AM, Konotchick T, Malay M, Maragos JE, Obura D, Pantos O, Paulay G, Richie M, Rohwer F, Schroeder RE, Walsh S, Jackson JBC, Knowlton N, Sala E. (2008) Baselines and Degradation of Coral Reefs in the Northern Line Islands. *Plos One.* 3 10.1371/journal.pone.0001548
